# Role of Saponins in Plant Defense Against Specialist Herbivores

**DOI:** 10.3390/molecules24112067

**Published:** 2019-05-30

**Authors:** Mubasher Hussain, Biswojit Debnath, Muhammad Qasim, Bamisope Steve Bamisile, Waqar Islam, Muhammad Salman Hameed, Liande Wang, Dongliang Qiu

**Affiliations:** 1 College of Horticulture, Fujian Agriculture and Forestry University, Fuzhou 35002, China; mubasherhussain05uaf@yahoo.com (M.H.); biswo26765@yahoo.com (B.D.); 2 State Key Laboratory of Ecological Pest Control for Fujian and Taiwan Crops, Fujian Agriculture and Forestry University, Fuzhou 350002, China; qasim_gill54@yahoo.com (M.Q.); bamisopebamisile@yahoo.com (B.S.B.); 3 College of Plant Protection, Fujian Agriculture and Forestry University, Fuzhou 350002, China; waqarislam@m.fafu.edu.cn (W.I.); mhameed@gudgk.edu.pk (M.S.H.); 4 Key Laboratory of Integrated Pest Management for Fujian-Taiwan Crops, Ministry of Agriculture, Fuzhou 350002, China; 5 Key Laboratory of Biopesticide and Chemical Biology, Ministry of Education, Fuzhou 350002, China; 6 Institute of Applied Ecology and Research Centre for Biodiversity and Eco-Safety, Fujian Agriculture and Forestry University, Fuzhou 350002, China; 7 Ministry of Agriculture Key Lab of Molecular Biology of Crop Pathogens and Insects, Institute of Insect Science, Zhejiang University, Hangzhou 3100058, China; 8 College of Geography, Fujian Normal University, Fuzhou 350007, China; 9 Faculty of Agricultural Sciences, Department of Plant Protection, Ghazi University, Dera Ghazi Khan 32200, Pakistan

**Keywords:** bioactive molecule, biological management, host plant resistance, plant immunity, plant secondary metabolites, triterpenoids

## Abstract

The diamondback moth (DBM), *Plutella xylostella* (Lepidoptera: Plutellidae) is a very destructive crucifer-specialized pest that has resulted in significant crop losses worldwide. DBM is well attracted to glucosinolates (which act as fingerprints and essential for herbivores in host plant recognition) containing crucifers such as wintercress, *Barbarea vulgaris* (Brassicaceae) despite poor larval survival on it due to high-to-low concentration of saponins and generally to other plants in the genus *Barbarea*. *B. vulgaris* build up resistance against DBM and other herbivorous insects using glucosinulates which are used in plant defense. Aside glucosinolates, *Barbarea* genus also contains triterpenoid saponins, which are toxic to insects and act as feeding deterrents for plant specialist herbivores (such as DBM). Previous studies have found interesting relationship between the host plant and secondary metabolite contents, which indicate that attraction or resistance to specialist herbivore DBM, is due to higher concentrations of glucosinolates and saponins in younger leaves in contrast to the older leaves of *Barbarea* genus. As a response to this phenomenon, herbivores as DBM has developed a strategy of defense against these plant biochemicals. Because there is a lack of full knowledge in understanding bioactive molecules (such as saponins) role in plant defense against plant herbivores. Thus, in this review, we discuss the role of secondary plant metabolites in plant defense mechanisms against the specialist herbivores. In the future, trials by plant breeders could aim at transferring these bioactive molecules against herbivore to cash crops.

## 1. Introduction

**Figure 1:**
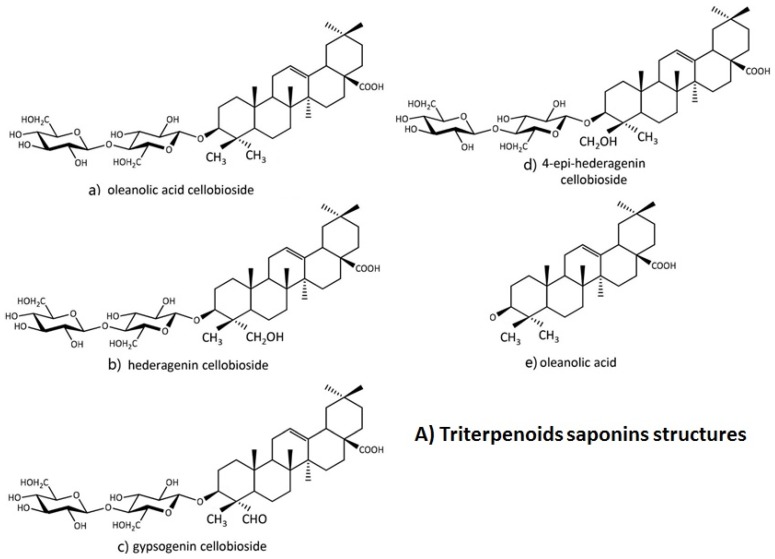
Triterpenoids saponins identified in *Barbarea vulgaris.*

The capacity of individual plant species to develop novel metabolites has been affirmed in charge of their imperviousness to plant herbivores. Plants have developed surprising diversity of substance protections against plant herbivores in light of bioactive mixtures of low atomic weight. A case of the bioactive mixtures utilized by plants in this regard are the triterpenoid saponins (Figure 1); which encourages plant immunity against a wide range of insect pests, pathogens, as well as other herbivores. 

Triterpenoid saponins are mostly found in dicotyledonous species whereas monocots mainly synthesis steroidal saponins. Some leguminous crops such as: Pea, sugar beet, soybeans, cowpea, asparagus, and capsicum peppers have been reported to contain saponins [1]. Saponins are considered one of the immeasurable and distinct groups of bio-plant items, and categorize secondary plant metabolites with particular natural properties [2,3]. Saponins content in plants is dynamic, and it influences various biotic stimuli that are related to pest attack, pathogenic infection, plant mutualistic symbioses with rhizobial bacteria and mycorrhizal fungi [2]. About over 200 different structures of saponins had so far been described [4]. Likewise, Khakimov, et al. [5] reported that blends of different chemical structures are accumulated by saponin producing plants. The biological activity of saponins can be attributed to the amphipathic properties of the constituting molecules, which consist of a hydrophobic triterpene or sterol backbone and a hydrophilic carbohydrate chain. Some saponins have potent biological activities that are influenced by other aspects of their structure. 

Saponins have been reported from different and unrelated plant families [5]. Whereas cereals are insufficient in saponins, aside from a few species of grass, such as *Panicum virgatum*, *Panicum coloratum*, and *Avena* spp. [6,7,8]. Additional gene families which have been reported to be involved in saponin biosynthesis and diversification are methyl and acyltransferases [9,10]. Aside crucifers, saponins occur constitutively in many other plant species as part of their defense system. For Brassicaceae, just a couple of species are known to yield saponins [11,12]. For example, the species from genus–*rea* {wintercress, *B. vulgaris* R. Br. (Brassicaceae)} are identified to create saponin; which are directly related to the plant defense against specialist herbivore, such as the diamondback moth (*Plutella xylostella*) (Lepidoptera: Plutellidae) [5]. *P. xylostella* is a typical crucifer specialist that is known worldwide as a severe pest of cruciferous crops, such as cabbage (*Brassica oleracea*) and oilseed rape (*Brassica napus*) [13,14]. 

Most of the glucosinolates–containing crucifers are all suitable hosts for the plant pest. The attractiveness of *P. xylostella* to these plant species is as a result of the glucosinolates content and its secondary products; such as isothiocyanates [15]. These compounds have been found to stimulate oviposition by *P. xylostella* adults, as well as, feeding by the larvae [16]. A similar observation has also been reported in cabbage butterfly (*Pieris rapae*); another crucifer specialist by Huang, Renwick and Sachdev-Gupta [15]. In another related report, a highly feeding deterrent activity to *P. xylostella* larvae was recorded in a chloroform extract of *B. vulgaris* leaves [17,18]. 

The management of *P. xylostella* has recorded minimum success as a result of its notorious ability to develop resistance to synthetic insecticides [13]. The ability of the pest to adapt plant secondary metabolites for host plant recognition, feeding, and oviposition stimulants has also been reported [19,20,21]. Moreover, inadequate knowledge of the biosynthetic paths and conducting systems of saponins has additionally complicated its application for pest control. However, the prospect of saponins modification as direct plant defense strategies against pests has offered alternative control measure for inclusion in an integrated pest management program for *P. xylostella*.

## 2. Plant Defense and Evolution

A variety of plants is susceptible to environmental disputes, but could not escape. In spite of this evident exposure, the Earth’s flora has developed to be highly abundant and diverse. It’s a reality that not all plants are entirely consumed, this could be as a result of top-down control [22], also to bottom-up mechanisms such as the direct defense of plants in response to herbivores [23]. Plants might play a major role in top-down control of herbivores by enrolling natural enemies of their enemies as an indirect defense [24]. Regarding a wide range of herbivores, direct plant defense mechanisms can demand structural adjustments, for example, trichomes, thistles, and silica bodies or assistance some other natural products. Furthermore, the auxiliary metabolites that potentially built up are lethal to herbivores, or attract the natural foes of the herbivores [25]. Disregarding their name, derivative metabolites have a vital impact on the chemical communication between plants and their surroundings. They are of basic significance for the appeal of pollinators (terpenes), protecting the plant as opposed to UV light (flavonoids), pathogens and herbivores (alkaloids, glucosinolates, saponins). The majority of plants comprises a significant range of plant derivatives [26]. From a developmental point, this range is mystified however even ineffectively understood. 

The reciprocal process of adaptation within plants and their insect herbivores was observed by Stahl [27], and he proposed that the synthetic mixtures may be included. These above thoughts were advanced by Ehrlich and Raven [23] to deliver a hypothetical background for the compound nature of insect and plant communications. They proposed a well-ordered biochemical co-advancement amongst plants and bugs. Unexpectedly, some herbivore species build up a resistance against biochemical compounds that are dangerous and distasteful to different insect pests. Gradually these biocompounds may possibly act as feeding stimulant or attractant for a particular insect, which has changed according to certain conditions and even utilizes some biochemicals as a guard, from the respective plant. It is useful for the insect pest as the plant constitutes a habitation which is limited for other generalist insect pests that are dissuaded by the biochemical compounds [28,29]. As a result, plants require new chemical admixtures to be ensured against these particular groups of insects. This procedure may bring about a proportional, well ordered “arms race” inside insect pests and its host plant, driving a wide range of biochemical-barrier mixtures [30].

## 3. Chemical Variety of Secondary Metabolites

Recently different ecological and evolutionary theories explain the chemical variety of secondary plant compounds. Generally, plants are required to be able to compete for the vast range of aboveground and belowground specialist herbivore. Consequently, they may incidentally be in a similar place; and compete with phytophagous arthropods, and other microorganisms, like virus, bacteria, as well as fungi [31,32,33]. As a result of insect pest’s diversity and the co-occurrence scope of bolstering plans, the plant requires mixtures of biochemicals for its defense. Thus, a wide range of biochemicals may give such protection [34]. Additionally, a variety of biochemical compounds is required by plants for producing strong physiological and biochemical effects to fight against different kinds of herbivores (see Table 1). Moreover, very lethal admixtures may have an adverse effect on many beneficial insects which are factually valuable for plants. Examples are pollinators and parasitoids [35,36]. Thus, the plant should have the capacity, to recognize phytophagous insect pests with comparable characters, as well as focus on its defense towards an exact body to maintain a strategic distance from such contrary impact on beneficial insects. In this way, a substantial diversity of chemicals would be required with a high specificity [37]. As a result, chemical variety is strongly motivated by the development of phytophagous insect pests. Since development is well on the way to request just a single or a couple of phytophagous insect pests, alternate phytophagous arthropod will remain prevented by the old biochemical. As an outcome, a compound has a capacity to protect, and it might be helpful to extend the biochemical range of a particular plant, in spite of substituting the old synthetic compounds with a recent chemical. Thus, the ethical force of the plant and phytophagous insect co-operations involves a distinct and dynamic arrangement of biochemical compounds.

## 4. Balance of Costs and Benefits by Formation of New Compounds

If a gene is changed in an individual plant, the fortune of this gene relies on how it affects the plant’s fitness. A change regarding mutation can be deleterious, neutral or beneficial. If mutations are deleterious they will quickly diminish, but on the other hand, useful ones will soon be changed in the population by natural selection. When the “new” and “old” gene are selectively neutral, polymorphisms can become balanced, and the selection keeps segregating alleles for extended periods of time [167]. Subsequent of the aggregate adjustment of expenses and advantages in the natural ecosystem of the plant portrays the variety among various groups of population and species in amount and kind of protection. Such modification can affect the competitiveness amongst genotypes and as a result the choice for a specific genotype [168]. Beside the undeniable advantages clarified in the previous, large amounts of protection, without enemy violence, are thought to be expensive [169,170]. The defense expenses are mostly visualized regarding the distribution of minimal assets from different vigor upgrading capacities inside a plant, for example, photosynthesis, development, as well as new generation [171,172]. Though, those expenses are not evident, as were assumed for plant biochemicals, especially volatiles along with a particular amount of terpenoids by Dicke and Sabelis [24], and Gershenzon [173]. Some defenses may demand ecological exchanges [168], so when supplies are distributed to protect against a particular phytophagous insect, it can decrease the vigor of the plant when harm triggered by other non-target phytophagous insect increments. Eventually, it is expensive when protection admixtures discourage advantageous living bodies, for example, crop pollinators and expected enemies of the phytophagous insect pests [174]. 

A diversity of plant defense chemical compounds can act as shields in contrast to insects, involving alkaloids, flavonoids, glucosinolates, and phenolic acids [175]. Mostly chemical compounds production is prompted by certain biotic or abiotic factors. Such a schematic arrangement is considered as fight against pathogens and frame insurance economically. Various chemical admixtures involved against insects are the saponins, which have distinctive chemical configurations commonly containing a triterpenoid and steroidal core with a differing quality of glycosylation structures. Saponins are presented in References [176,177] particular 100 various plant categories, even though they mostly are general in species from distinct families or genre, for example, Leguminosae and Liliaceae [178]. Saponins are acquired independent from outside signals and lead to the innate immunity, so named as hypo anticipations because they introduced in individual plants. 

The positive aspect of cumulating saponins is primary protection, which is not just for huge measures of vitality, as well as for pathogens to develop mod additionally that it makes feasible for pathogens to develop moderation. It voided when saponin antecedents cumulate and saponin stuff raises resultantly chemical changes of precursor molecules, which incited by pathogenic contamination [179]. Perhaps, the saponin substance may build ideally to the limited quantity due to the chemical response of deposited precursors for biochemical compound safety system or because of pathogen given debasement [180,181]. 

In the beginning, several studies data on the specific activity of saponins against insects were limited to leguminous origins and extracts [182]. Hostettmann and Marston [183] indicated that several high saponin plant parts from various families, including Aquifoliaceae, Theaceae, as well as Leguminosae, are resistant to insects. Recently, a lot of studies showing the structural activity of concentrated or pure saponin fractions against insects have widely elaborated, and have influenced insects such as aphids, beetles, caterpillars, and flies [1,184]. Nevertheless, the relevant studies of the consequences of various saponins from different origins against insects of different feeding differentiation are still limited.

The behavior of insects changes with individual components of host food, some nutrients attract the insects, while others repel. Hence, plants can synthesize some substances that are important for their significant exercises, while the auxiliary metabolites are included during the time spent co-development amongst plants and other living organisms, for example, insects [185,186]. *P. xylostella* is a serious pest of cruciferous crops with a cosmopolitan distribution [187]. *P. xylostella* has developed resistance to existing chemical insecticides including the *Bt* toxin [188], making it increasingly difficult to control [189]. The capacity of *P. xylostella* to quickly create imperviousness to insecticides, joined with typically ecological and suitability risks, have fortified enthusiasm for optional controlling systems, for example, trap crops [190]. A trap crop proposed for *P. xylostella* is wintercress, *B. vulgaris* [191,192,193,194]. It is a biennial or short-lived perennial plant native to temperate regions worldwide [195]. According to the findings of Shinoda, Nagao, Nakayama, Serizawa, Koshioka, Okabe and Kawai [16], the response of *P. xylostella* larvae is to be suspected that there is a feeding-deterrent in a crucifer-*B. vulgaris*. They recorded an adverse effect of the plant volatile compounds on the specialist pest larvae, as the feeding rate of larvae of *P. xylostella* was reduced on the plant. The feeding deterrent was isolated from *B. vulgaris* leaves and was identified through the structure to be a monodesmosidic triterpenoid saponin.

## 5. Larval Feeding Preference and Adult Oviposition Behavior

Larval feeding choice and adult oviposition for younger leaves when contrasted with more seasoned leaves of a specific accommodating plant is a general pattern common with numerous phytophagous insects, particularly in connoisseurs, encompassing *P. xylostella* [196]. Whenever *P. xylostella* adults have an option of *B. vulgaris* and different cruciferous crops, despite the fact *P. xylostella* larvae cannot continue their lives on a limited range of *B. vulgaris*, as such as plants being much supportive for oviposition of *P. xylostella* adults [16,197]. This non survivorship is thought to be as a result of saponins [196]. 

### 5.1. P. xylostella Larval Survival on Cotyledons and True Leaves within the Same Plant

Cotyledons represent the capacity of food storage for the improvement of plant, which is the primary photosynthetic network for the plant after germination [198], cotyledons of brassicaceous plants contain varying contents of glucosinolates [199,200]. In *Barbarea* plants, glucosinolates that might protect the plants against generalist herbivores, were present in the cotyledons, while saponins, which could defend the plant against specialist herbivores like *P. xylostella.* Similarly, some saponins were not present in cotyledons, indicating that there might be some other biochemical compounds which are responsible for plant defense against herbivores.

### 5.2. Saponins Presentation in B. Vulgaris Var Arcuata (Isolation and Identification)

The isolation and identification of a triterpenoid saponin, from the leaves of *B. vulgaris*, which strongly deters feeding of *P. xylostella* larvae and also the oleane type saponin was studied by Shinoda, et al. [201]. Nielsen, et al. [202]) and Augustin, et al [4] found five triterpenoid saponins in *B. vulgaris* namely; 3-O-cellobiosyl-hederagenin (hederagenin cellobioside), 3-O-cellobiosyl-oleanoic acid (oleanolic acid), 3-O-cellobiosyl-gypsogenin (gypsogenincellobioside), 3-O-cellobiosylcochalic acid (cochalic acid cellobioside) and 3-O-cellobiosyl-4-epihederagenin (4-epihegragenin cellobioside) Hederagenin cellobioside and oleanolic acid (Figure 1), which make *B. vulgaris* resistant to *P. xylostella* and are correlated with deterrence of adult *P. xylostella* females [4,52,203]. Shinoda, et al [16] discovered that this is not only the first feeding deterrent to *P. xylostella* found in the family Brassicaceae, but also the first oleanane-type saponin found in this family. So, advance clarification of the chemical configuration of saponins could enhance the development of hydrophobic analogs which may be characterized as fascinating insecticides and herbicides, which potentially required for ecologically more suitable than present synthetic pesticide and herbicides.

## 6. Biological Significance of Saponins

Saponins are biochemical compounds or otherwise depicted as natural products, which have an extensive spectrum of natural performances. Numerous biological roles have been indicated for various saponins, including anti-inflammatory allelopathic action, anti-carcinogenic, mitigating cell reinforcement, heamolytic, hypocholesterolemic resistance stimulators, cell layer permeabilizing characteristics, as well as can influence feeding behavior, development, and cause mortality, development hindrance, limit the insects’ productiveness and protection against insects and other micro-organisms. 

### 6.1. Saponins Interference with the Feeding Behavior

Some previous reports are available indicating the inability of insect pests larvae to attack Brassicaceae species (*B. vulgaris*) due to triterpene (saponin), along with two sugars at the position of C3, which restrain the prosecution of the food uptake [201]. Saponins also showed strong effects against other pathogens like fungi mollusks certain bacteria and viruses. In general, it is believed that such biochemicals operate crucially in the plant protection against biotic, as well as abiotic factors, as reported from soybean saponins, which had shown detrimental effects against *Tribolium castaneum*, *Bufo viridis* and *Lebistes reticulatus*. Similarly, saponins were also observed to check the cholinesterases, as well as the proteolytic drive of other enzymes, like trypsin, chymotrypsin and papain, which leads towards non-specific communication with other protein. Moreover, some studies reported that *Quillaja Saponaria* saponins induce fatality in living insects, and a potent cytotoxic activity on other insects like *Drosophila melanogaster* cells [204].

### 6.2. Saponins Effects on Protein Digestion

The toxicity of saponins to various organisms linked to their interaction with biological membranes. Some saponins form complexes with proteins [205] and by this action, they apparently inhibit proteinases and affect digestion in insect gut [204,206,207]. The capability of saponins to penetrate the cell membrane and to induce apoptosis makes saponins cytotoxic to lepidopteran cells [204].

### 6.3. Enterotoxicity

Saponins are a group of steroidal or triterpenoid secondary plant metabolites, with divergent biological activities [208,209,210], they are responsible for plant defense against antagonists; such as mollusks, pathogens and insects [211,212]. The combination of hydrophilic sugars and hydrophobic sapogenin enable saponins to incorporate into biological membranes. Toxicity of saponins to different organisms seems to be related to their interaction with biological membranes and might be related to their soap-like properties. As a result, detoxification of saponins is probably regarded as enzymatic hydrolysis of the glycosidic bonds, as already produced for fungi [213,214]. 

Many crucifer specialist insects, such as *Pieris brassicae* and *Pieris rapae* and *Pieris nemorum* with R-genes, are insusceptible to the defenses of *B. vulgaris*. By finding out the structures of saponins in *B. vulgaris* [16,215] has allowed for investigations into the mechanism by which these in susceptible insects can deal with the potentially toxic saponins. Badenes-Perez, Reichelt, Gershenzon and Heckel [196] reported that the struggle of *B. vulgaris* to the diamondback moth (DBM) is prompted by two different saponins; I) 3-0-b-cellobiosylhederagenin and II) 3-0-cellobiosyloleanolic acid, which prevents the feeding of *P. xylostella*. Likewise, it had been reported that the combination of feeding deterrents showed feeding deterrent habituation in other insects and the combination of saponins I and II may also slow down feeding deterrent habituation in *P. xylostella*. Nevertheless, saponins I and II contain similar chemical structures; cross habituation might be easier as compared to compounds with different chemical structures, which also indicate the synthesis of saponin-II could be after that of saponin-I [216,217,218,219]. 

Idris and Grafius [220] and Badenes-Pérez, et al. [221] showed that a small percentage of larvae of a *P. xylostella* population collected from the field were able to survive on *B. vulgaris*, even though they did not report the concentration of saponins in these plants. Further researches are required to verify in any case being feeding deterrents, saponins I and II, might have a toxic effect on *P. xylostella* larvae. Badenes-Perez, Reichelt, Gershenzon and Heckel [196] observed that continuous feeding of neonates of *P. xylostella* usually on resistant *B. vulgaris*, results in feeding signs [192]. 

Dissimilarly to glucosinolates, saponins I and II do not have all the earmarks of being expressed on the leaf covering of *Barbarea* [222]. Therefore, it is probably that neonates of *P. xylostella* encounter glucosinolates on the leaf surface and start feeding, while feeding is reduced when insects come into contact with the saponins in the leaf tissue. Likewise saponins I and II, other saponins have been segregated from P-type *B. vulgaris* var. *arcuata*, which are responsible for the resistance of this plant to *P. nemorum* [53,223]. Given the similarity in the resistance mechanisms of G-type *B. vulgaris* var. *arcuata* to both *P. nemorum* and *P. xylostella*, these saponins might be required in the resistance of *Barbarea* to *P. xylostella.* Saponins display higher toxicity, even though the precise mode of action of saponins remains unresolved, it was reported by Badenes-Perez, et al. [196] that saponins specifically target pest insects: Both the continuous insect cells and the primary midgut cells of *Spodoptera littoralis* showed high sensitivity to *Q. saponaria* saponin. The phenomenon behind such synergistic mechanisms are unknown, but may include the ability of one biochemical to inhibit the detoxification of other components or to up regulate the absorption of others from the gut.

More significantly, the saponins can cause great and quick in vivo enterotoxin results on the larvae of *S. littoralis*, and with contents likewise those that can be presented in nature. Therefore, saponins showed substantial evidence for the potency in the control of pest insects, especially insect midgut epithelium as the primary target tissue. So, the insect midgut is an attractive target, as any damaging effect on the midgut epithelial cells will result in starvation, leading towards slow insect mortality. As this component is not the same in midgut cells as the approach of *Bacillus thuringiensis* (Bt), it can likewise be of assistance in the management of imperviousness to *Bt*. Furthermore, as aphids are not perceptive to the poisons of *Bt*, all observations propose that saponins may represent a noteworthy outcome in developing new, substitute, environmentally favorable aphid control agents amongst integrated pest management.

## 7. Limits of the Use of Saponins in Pest Management Control

Some saponins have heamolytic and cytotoxic effects which have the potential of inhibiting the protease activity. Due to this constraint, it is difficult to apply in the field, as they might also be toxic to humans. The saponins function to protect host plant and to discourage phytophagous insects usually is explained according to their performance in the body of the exposed organism, such as less food consumption, obstructions as well other poisons [224,225]. Mostly, saponins are known as disincentives against insect pests, but their mode of action is yet relatively obscure, however it is identified to interrupt cell sheets [213,226]. 

Moreover, it was assumed with respect to insects that insect resistance, on the base of ecdysteroid receptor complex (EcR), may be due to particular steroidal saponins, which have resemblance with 20-hydroxyecdysone (molting hormone) [227,228]. Even though, the saponins performance was not supported by real resistance reaction to EcR communication, yet rather than loss of cellular unity considerably, due to the pervasion of the insect cell layer, as described by De Geyter, Swevers, Caccia, Geelen and Smagghe [204]. Plant-derived triterpenoid and steroidal saponins are very promising for the development of botanical insecticides. Aside from cellular poisoning quality, saponins additionally exhibited hindrance or anti-feedant drive against herbivores, especially insects. In a previous study, it was reported that saponins (aginosid) extracted from leek (*Allium porrum*) caused a noteworthy obstruction in response to two Lepidopteran insect pests; *Peridroma saucia* and *Mamestra configurata* [229], as well as in sucking insect pests [230]. In another related study, extracts from the roots of *Saponaria officinalis* induced a reduction in the rate of oviposition by females of *Tetranychus urticae* [231]. The plant extracts were found to contain a mix of various saponins [232,233,234] which are suggested to be responsible for the acaricidal efficacy. However, the mechanism of action on mites needs to be further explored. 

## 8. Conclusions and Recommendations

Glucosinolates and saponins play an important role in the plant defense against specialist herbivores. Preliminary data on the saponins’ performance was constrained to reports of leguminous reserves as well other by-products [182]. Thus, comparative studies on the role of saponins are still limited. The chemical basis of previously reported flea beetle resistance in the G-type of *B. vulgaris* var. *arcuata* is unknown, but resistance is not correlated to glucosinolates or glucosinolate levels [235]. Resistance may be due to the occurrence of a triterpenoid saponin, which made resistant to *B. vulgaris* against DBM [16]. Development in the interpretation of saponins biosynthetic system has been obstructed due to a distinctive molecular configuration along with the complication of enzymes, related to two major superfamilies, such as I) cytochrome (P450) and II) glycosyltransferase (GT). The greater part of the *Allium* and *Calamus* species consist of saponins, which have a crucial role in health; as such saponins are responsible to decrease the level of garlic cholesterol, as well as enhance the anti-fungal function of garlic [236,237]. They display higher toxicity, even though the precise mode of action of saponins remains unresolved. It has been reported by Badenes-Perez, et al. [196] that saponins from *Q. saponaria* approach *S. littoralis* directly by affecting consistent insect midgut cells. It might be exemplified significant results in developing new, substitute, environmentally favorable control agents amongst integrated pest management. 

As the discovery of plant defense chemicals continues at its present rapid pace, the present studies discussed above represent the role of plant secondary metabolites in plant defense against herbivores. Given the complex chemical structures of plants, which are not easy to fully understand, play the actual role in defense mechanism (adaptations or counteradaptations) in plant–herbivore interactions. Thus, the structure activity studies of saponins as deterrents for specialist herbivore (*such as P. xylostella*), therefore, are useful for the deeper understanding of the components and the systems concerned with insect resistance. However, a targeted isolation of these insect repellants will elucidate their structures. Therefore, the improvement of hydrophobic analogs might be regulated by a particular chemical structure of saponins, which may be characterized as interesting chemical sprays, for a particular range of plants, and are (potentially) more natural, compared to the present synthetic herbicide used against herbivores.

## Figures and Tables

**Table 1:** Biochemical compounds responsible for plant defense against herbivores.

Family	Plant	Secondary Metabolite Form	Reference
Aceraceae	*Acer velutinum*	Td.S	[38]
Agavaceae	*Agave sisalana*	S.A	[39]
Amaranthaceae	*Achyranthes bidentata*	Te.S; Bidentatoside II and chikusetsusaponin V methyl ester.	[40]
	*Chenopodium quinoa*	Td.S	[1,41]
	*Beta vulgaris*	Td.S	[42,43]
Apiaceae	*Steganotaenia araliacea*	Td.S	[44]
Aquifoliaceae	*Ilex opaca*		[45]
Araliaceae	*Panax ginseng*	Te.S; Ginsenosides, glycosides of triterpenoid aglycones	[46,47]
Asparagaceae	*Yucca schidigera*	S.S	[48,49]
	*Asparagus officinalis*	S.S	[1,41]
Asteraceae	*Atractylis flava*	Td.S	[50]
Brassicacea	*B. vulgaris*	Td.S; hederagenincellobioside, oleanolic acid cellobioside, epihederagenincellobioside, and gypsogenincellobioside	[4,51,52,53]
Campanulaceae	*Platycodon grandiflorum*	Td.S	[54]
Caryophyllaceae	*Saponaria officinalis*	Td.S	[55,56,57]
Columelliaceae	*Desfontainia spinose*	Triterpenoids	[58]
Combretaceae	*Combretum nigricans*	Cytotoxic pentacyclic triterpenes	[59]
Compositae	*Aster auriculatus*	Td.S	[60]
	*As. tataricus*	Triterpene glycoside	[61]
	*As. ageratoides*	Td.S	[62]
	*As. batagensis*	Td.S	[63,64,65]
	*As. bellidiastrum*	Td.S	[66,67]
	*As. lingulatus*	Td.S	[68,69]
	*As. scaber*	Td.S	[70]
	*As. sedifolius*	Oleane-type saponins; Astersedifolioside A, B and C	[71]
	*As. yunnamensis*	Td.S	[64,72,73]
Cucurbitaceae	*Gynostemma pentaphyllum*	Gypenosides	[74,75]
	*Momordica charantia*	Td.S	[76]
Dioscoreae	*Dioscorea* spp.	Te.S, Dioscin	[77,78]
Fabaceae	*Glycyrrhiza* spp.	Glycyrrhizin; Td.S	[79,80]
	*Medicago sativa*	Td.S	[81,82,83]
	*Desmodium adscendens*	Td.S	[84,85]
Flacourtiaceae	*Aphloia madagascariensis*	Te.S	[86]
Flacourtiaceae	*Aphloia theiformis*	Te.S	[87]
Hippocastanaceae	*Aesculus* spp.	Td.S; Escins Polyhydroxyoleanene pentacyclic triterpenoid saponins; Aesculiosides	[88,89,90,91,92]
Lamiaceae	*Salvia staminea*	Td.S, *salvistamineol*	[93]
Lecythidaceae	*Petersianthus macrocarpus*	Td.S	[94,95]
	*Barringtonia acutangula*	Monodesmosidic glucuronide saponins; Barringtosides A, B and C	[96]
Liliaceae	*Allium aflatunense*	S.S	[97,98]
	*A. albanum*	S.S	[99]
	*A. albiflorus*	S.S	[100]
	*A. albopilosum*	S.G	[101]
	*A. ampeloprasum*	S.S	[102,103,104]
	*A. ascalonicum*	S.S	[105]
	*A. cepa*	S.S; furostanol saponins, ceposide A, B, and C	[106,107,108,109]
	*A. chinense*	S.S	[110,111,112]
	*A. elburzense*	S.S	[113]
	*A. erubescens*	S.S	[114,115]
	*A. fistulosum*	S.S	[116]
	*A. giganteum*	S.S	[97,117,118,119]
	*A. jesdianum*	S.G	[120]
	*A. karataviense*	S.S	[121,122]
	*A. macleanii*	S.G	[123]
	*A. macrostemon*	Furostanol glycosides	[124]
	*A. narcissiflorum*	S.S	[125,126,127]
	*A. nutans*	S.S	[128,129]
	*A. ostrowskianum*	S.G	[101]
	*A. porrum*	Spirostane-type saponin	[130,131,132]
	*A. sativum*	S.S	[111,133,134,135]
	*A. schubertii*	S.S	[136]
	*A. sphaerosephalon*	Furostanol saponin	[137]
	*A. senescens*	S.G	[123]
	*A. triquetrum*	S.S	[138]
	*A. tuberosum*	S.S	[139,140]
	*A. turcomanicum*	S.S	[141]
	*A. vineale*	Molluscicidal saponins	[142]
	*A. waldstenii*	Steroids of spirostan and furostan series	[115]
Loganiaceae	*Antonia ovata*	Td.S	[143]
Myrsinaceae	*Myrsine pellucida*	Te.S	[144]
	*Tapeinosperma clethroides*	Glucuronide saponins: Desacyl-jegosaponin, desacylboninsaponin A, and sakuraso-saponin	[145,146]
Nyctaginaceae	*Pisonia umbellifera*	Oleanolic acid saponins and Seco-glycopyranosyl moiety.	[147]
Phyllanthaceae	*Glochidion eriocarpum*	Cytotoxic oleane-type triterpene saponins	[148]
Phytolaccaceae	*Phytolacca bogotensis*	Te.S	[149]
Poaceae	*Avena sativa*	S.S	[1]
Quillajaceae	*Quillaja saponaria*	Te.S	[150,151]
Ranunculaceae	*Anemone flaccida*	Te.S	[152,153]
Rhamnaceae	*Ziziphus joazeiro*	Triterpenicaglycone	[39]
Rosaceae	*Rosa laevigata*	Triterpene glucosides	[154]
Sapindaceae	*Smelophyllum capense*	Te.S	[155]
	*Filicium decipiens*	Te.S	[156]
	*Harpullia cupanioides*	Triterpenoïdes	[157,158]
	*Sapindus mukorossi*		[159]
Sapotaceae	*Tridesmostemon claessenssi*	Tridesmosaponin A and B	[160]
	*Gambeya boukokoensis*	Gamboukokoensides A and B	[161]
	*Mimusops* spp.	Td.S	[162]
Solanaceae	*Solanum tuberosum*	S.S	[1]
	*S. melongena*	S.S	[1,41]
	*Capsicum species*	S.S; four glucose moieties and three glucose moieties	[1,163]
Symplocaceae	*Symplocos chinensis*	Td.S	[164,165,166]
Theaceae	*Camellia sinensis*	Td.S	[41]

S.A = Steroidal aglycone; S.S = Steroid saponins; S.G = Steroidal glycosides; St.S = Steroidal saponins; Td.S = Triterpenoid Saponins; Te.S = Triterpene saponins
